# Intraductal papillary neoplasm of the bile duct presenting with hepatogastric fistula: a case report and literature review

**DOI:** 10.3389/fonc.2023.1193918

**Published:** 2023-05-19

**Authors:** Wen-Hui Chan, Chien-Ming Chen, Shang-Yu Wang, Ren-Chin Wu, Tse-Ching Chen, Hao-Kang Lee, Cheng-Hui Lin, Chun-Nan Yeh

**Affiliations:** ^1^ Department of Medical Imaging and Intervention, Chang Gung Memorial Hospital at Linkou, and Chang Gung University College of Medicine, Taoyuan, Taiwan; ^2^ Department of Surgery, Chang Gung Memorial Hospital at Linkou, and Chang Gung University College of Medicine, Taoyuan, Taiwan; ^3^ Department of Pathology, Chang Gung Memorial Hospital at Linkou and Chang Gung University, Taoyuan, Taiwan; ^4^ Department of Gastroenterology and Hepatology, Chang Gung Memorial Hospital at Linkou, Taoyuan, Taiwan

**Keywords:** intraductal papillary neoplasm of the bile duct, hepatogastric fistula, cholangiocarcinoma, gastrectomy, hepatectomy

## Abstract

Intraductal papillary neoplasm of the bile duct (IPNB) is an uncommon entity characterized by papillary growth within the bile duct lumen. IPNB is regarded as a biliary counterpart of intraductal papillary mucinous neoplasm of the pancreas, which sometimes complicates with fistula formation to adjacent organs, mainly due to high-pressure related erosion from mucin-filled ducts. However, fistula formation from IPNB is quite rare. Here we report a case of IPNB complicated with hepatogastric fistula. Abdominal computed tomography (CT) and magnetic resonance imaging (MRI) revealed disproportional dilatation of left intrahepatic duct with intraluminal soft tissue nodules and fistulous connections to gastric high body. Endoscopy revealed ulcers with two fistulous orifices at upper gastric body. The patient underwent left hepatectomy with gastric wedge resection. Histopathology examination revealed IPNB with invasive cholangiocarcinoma, directly invading to gastric wall leading to hepatogastric fistula. In summary, we have presented the clinical, imaging and pathological findings, along with a comprehensive review of relevant literature, in order to enhance the understanding of this rare condition.

## Introduction

Intraductal papillary neoplasm of the bile duct (IPNB) is defined as a papillary or villous neoplasm occurring in the bile duct by the latest World Health Organization (WHO) classification (1). The occurrence of IPNB exhibits significant geographic diversity, with the most frequent cases being documented in Eastern Asian nations, which is likely due to the endemic presence of hepatolithiasis (2). IPNB encompass a disease spectrum from benign intraepithelial neoplasm to malignant cholangiocarcinoma (3, 4). According to a meta-analysis, invasive tumors were present in at least 43% of surgical specimens of IPNB, and individuals with such tumors had a notably poorer prognosis in comparison to those without evidence of invasion (5). Consequently, early surgical intervention is necessary in patients presenting with clinical and radiological evidence of IPNB to impede disease progression (5).

IPNB is usually considered as a biliary counterpart of pancreatic intraductal papillary mucinous neoplasm (IPMN-P), with the affected bile ducts in IPNB present with marked dilatation caused by mucin hypersecretion. Pancreatic IPMN infrequently fistulate into adjacent organs, such as the duodenum, stomach, and bile duct, with reported incidence ranging from 1.9% to 6.6% (6, 7). The occurrence of fistula penetrating to neighboring organs by IPNB is even rarer compared with IPMN-P, with only few case reports in the literature (8–14). In this study, we report a case with IPNB complicated by fistulous connections of left lobe liver and gastric body. We summarize the clinical, imaging, and pathology findings and perform a literature review to improve the understanding of this rare complication.

## Case presentation

A 72-year-old male presented with epigastralgia and body weight loss for half a year. The patient did not have any major relevant medical history. On physical examination, abdomen was soft and flat without tenderness. The complete blood counts were as follows: white blood cell count of 6400 uL, hemoglobin of 12.5 g/dL, and platelet count of 221000/uL. The blood chemistry panel revealed normal direct type and total bilirubin, alkaline phosphatase, liver enzymes including aspartate aminotransferase and alanine aminotransferase. Serum tumor markers showed elevated carcinoembryonic antigen (CEA) of 168 ng/mL, normal alpha-fetoprotein (AFP) and CA19-9.

**Figure 1:**
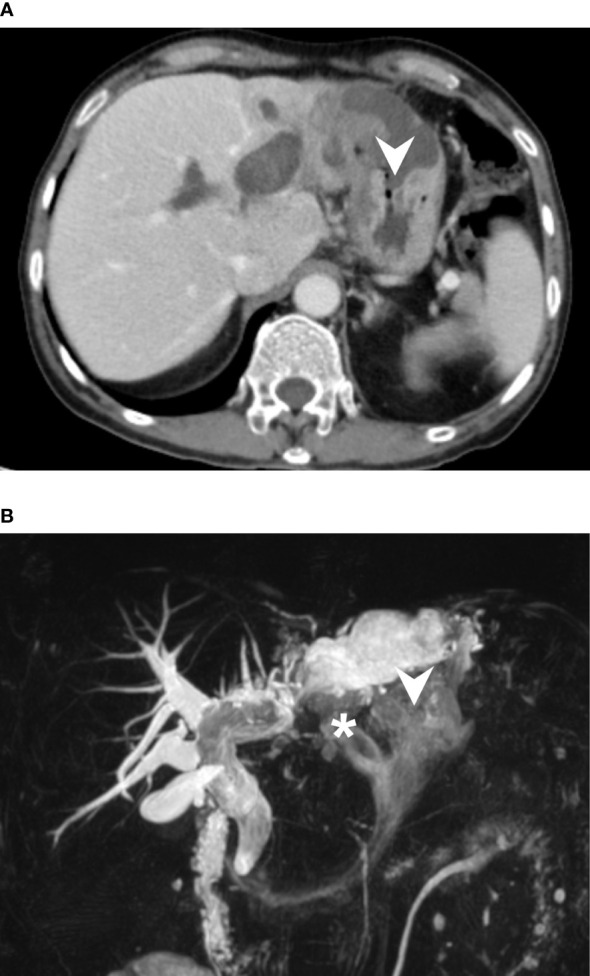
**(A)** CT axial view revealed a complex mass with cystic component and irregular solid part at S3 of liver. The mass was found to be invading gastric body directly (arrowhead). In addition, there was marked dilatation of right IHDs. **(B)** Magnetic resonance cholangiopancreatography (MRCP) demonstrated diffuse dilatation of intrahepatic and extra-hepatic bile ducts, likely caused by mucous accumulation, which is a signature of mucin-producing IPNB. The images clearly showed the biliary-gastric fistulae (indicated by arrowhead and asterisk). Notably, intraluminal filling defects were found in left IHD and CBD, which may represent intraluminal papillary tumors or mucobilia. However, intraoperative choledochoscopy ruled out papillary tumor growth in the CBD.

**Figure 2:**
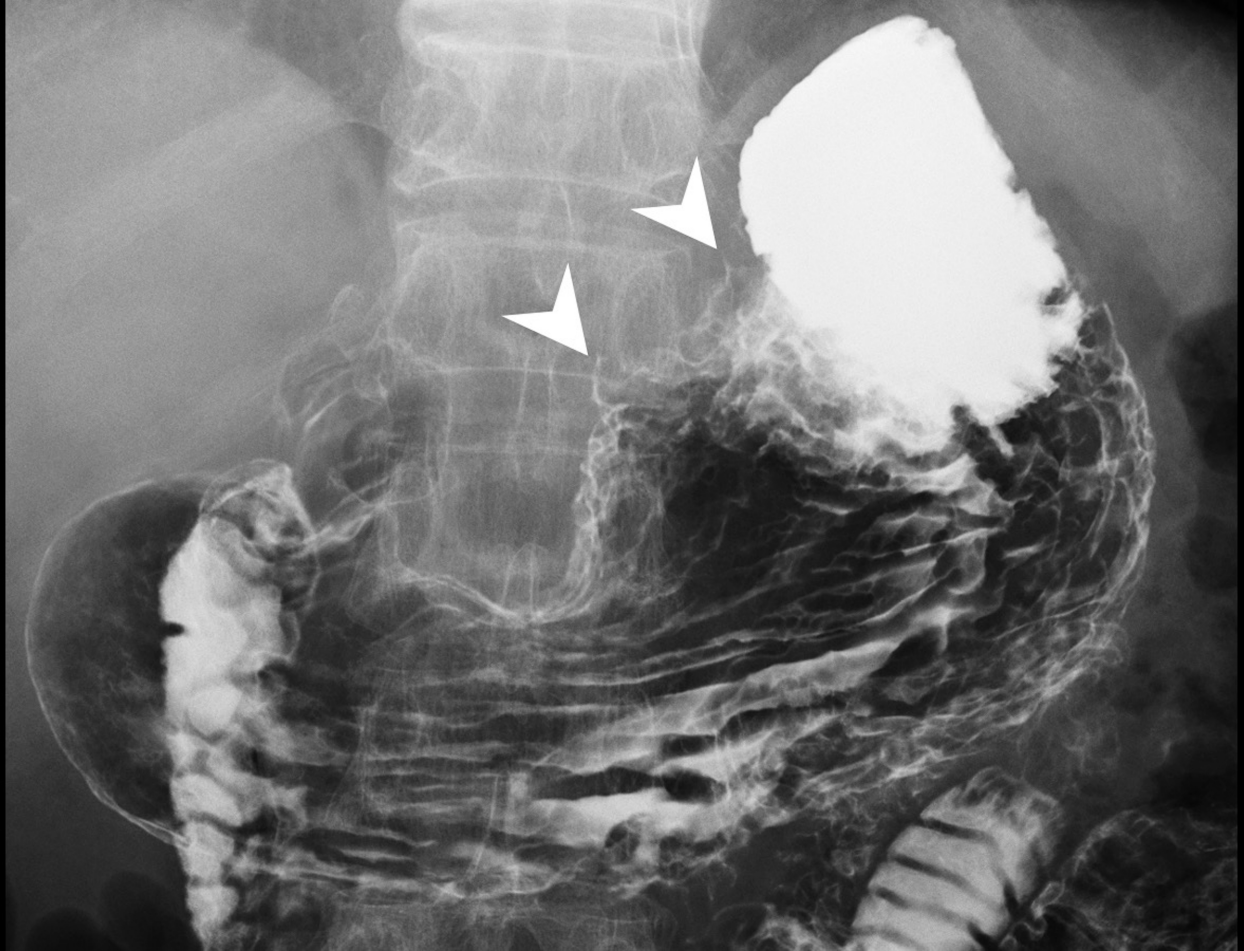
Upper GI barium study showed incomplete distention of lesser curvature side of gastric high body with mucosal irregularities, as well as two spiculations (arrowhead), probably related to fistula tracts. No definite extravasation of barium meal was found during the study.

**Figure 3:**
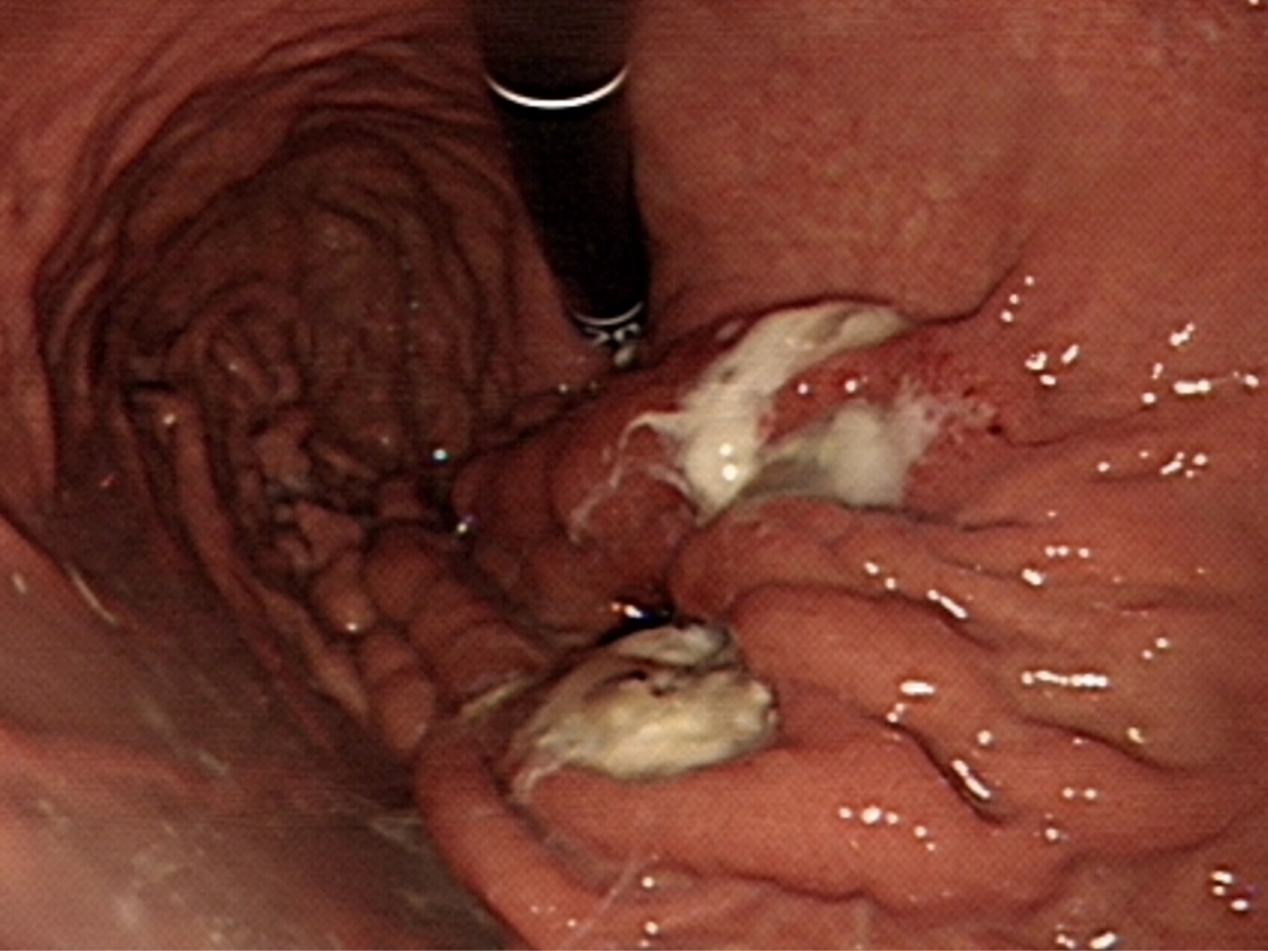
Endoscopy demonstrated two ulcers at lesser curvature side of the anterior wall of gastric high body near cardia. Upon closer evaluation, suspicious fistulous orifices exude mucin content.

Abdominal computed tomography (CT) revealed diffuse dilatation of bilateral intrahepatic ducts (IHD) and common bile duct (CBD), with a markedly dilated left IHD and relative sparing of the bile duct in caudate lobe. Presence of a complex mass at segment 3 with poorly enhancing mural nodules and indistinct margin between liver and gastric body (**Figure 1A**). Magnetic resonance imaging (MRI) showed marked dilatation of left IHDs with intraluminal soft tissue filling defect and fistulous connections to gastric high body. (**Figure 1B**). Upper gastrointestinal series demonstrated irregular mucosa with spiculated foci at lesser curvature side of upper gastric body (**Figure 2**). Gastroduodenoscopy revealed ulcers at lesser curvature side of upper gastric body, with exuding whitish mucous (**Figure 3**).

During laparotomy, intraductal papillary tumors located in left intrahepatic ducts were discovered to form fistula tracts with the anterior wall of stomach. A left hepatectomy with gastric wedge resection was performed. A frozen section specimen of the initial bile duct margin revealed atypical glandular cells, which prompted an additional resection of the remnant duct up to 3 mm, with the final bile duct margin negative of malignant cells. Lymphadenectomy of group 1, 3, 8 and 12 lymph nodes was also performed. Due to concerns about intraluminal tumor spread, intraoperative choledochoscopy was conducted down to the Ampulla of Vater, revealing no tumor growth in common hepatic duct or common bile duct, and up to the third order of the right intrahepatic ducts, revealing no tumor growth in the right intrahepatic ducts. The total operative time was 7 hours 35 minutes and blood loss was 1800 mL.

**Figure 4:**
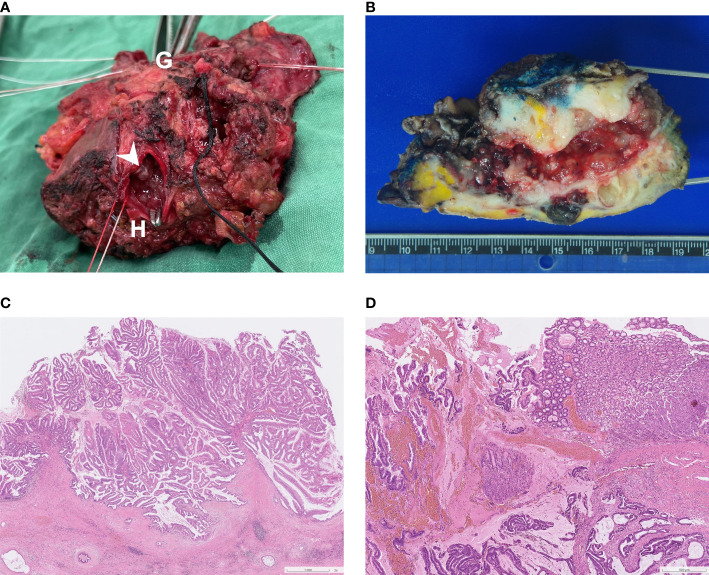
**(A)** Surgical specimen: A direct fistulous connection was found between left IHD to stomach. To confirm this, a forceps was inserted from the orifice of stomach (indicated by “G”) to left IHD (indicated by “H”). Several intraductal papillary tumors were found from the cut surface of left IHD (arrowhead). **(B)** Macroscopic findings of the cut surface: the specimen contains left hepatectomy (on the right) and wedge gastrectomy (on the left), with numerous whitish intraductal papillary excrescences and a hepatogastric fistula. Invasive carcinoma with periductal infiltrating growth pattern around the fistula track is noted. **(C)** Photomicrograph (hematoxylin and eosin stain, 2x objective lens) shows an intraductal polypoid neoplasm with florid proliferation of papillary fronds, which was characterized by fibrovascular cores covered by mixed intestinal and gastric-type epithelia. **(D)** Photomicrograph (hematoxylin and eosin stain, 4x objective lens) of the fistula (gastric end): Along the fistulous tract are mucin admixed with papillary fronds of tumor cells and neoplastic glands infiltrating in a desmoplastic stroma. Gastric mucosa is present on the right upper corner.

Upon gross inspection of the hepatectomy specimen, it was discovered that there were direct fistulous connections between left IHD and the stomach. Papillary tumors were also found on the cut surface of left IHD (**Figure 4A**). Macroscopically, the specimen demonstrated a mucinous tumor in left IHD with fistulous connection to gastric high body (**Figure 4B**). Microscopic histologic exam showed intraductal papillary neoplasm of bile duct (**Figure 4C**) with associated cholangiocarcinoma, as well as direct transmural invasion to gastric wall with fistula formation (**Figure 4D**). The bile duct margin of the hepatectomy specimen was involved by high-grade intraepithelial neoplasia while the final bile duct margin was negative. Six lymph nodes were retrieved from group 1, 3, 8 and 12, and the histopathological examination showed no evidence of malignancy.

Intraoperative bile duct culture yielded Escherichia coli and Enterococcus faecalis. Therefore, prophylactic antibiotic therapy was given to prevent postoperative surgical site infection. Fortunately, the patient had an uneventful postoperative course and was discharged from hospital on the 14^th^ postoperative day. Three months after the operation, a follow-up choledochoscopic exam was conducted through the T- tube. The results showed that the common bile duct and right hepatic ducts had a normal mucosal pattern and no intraductal papillary tumor or choledocholithiasis was detected. He remained at disease free status during 6-month follow-up period and was currently undergoing follow-up observation at outpatient clinic.

## Discussion

Intraductal papillary neoplasm of bile duct may involve intrahepatic, perihilar or distal bile duct (4, 5). The clinical and pathological manifestations of IPNB can differ widely, spanning from pre-invasive low to high grade intraepithelial neoplasm to invasive cholangiocarcinoma, and with variable amount of excessive mucin-production (5). IPNB is considered the biliary counterpart of pancreatic intraductal papillary mucinous neoplasm (3) (15), although the malignancy rate of IPNB is considerably higher than pancreatic IPMN (16). The 2019 WHO classification subclassified IPNB into type 1 (so-called IPNBs) and type 2 (narrow-sense papillary cholangiocarcinomas) (1). Type 1 IPNB is more frequently found in intrahepatic ducts and presents with fewer invasive components, while type 2 IPNB is more frequently found in extrahepatic ducts and demonstrates more complex papillary structures (1). Furthermore, a recent Japanese case series used a scoring system involving six pathological features (location, mucin secretion, histological architecture, etc.) to delineate a continuous disease spectrum from type 1, type Unclassifiable, and type 2, with significantly better 5-year survival rate in patients with type 1 tumors than patients with type Unclassifiable and type 2 tumors (17). IPNB may spread superficially *via* biliary mucosa, therefore thorough diagnosis with cross-sectional imaging (CT, MRI) and cholangioscopy is crucial for accurate localization (3, 18, 19).

Spontaneous biliary-enteric fistula is an abnormal communication of bile duct and gastrointestinal tract, with leading cause of recurrent cholelithiasis and cholangitis, perforated peptic ulcer and less commonly neoplastic infiltration from bile duct or gastrointestinal cancers (20). Fistula formation between bile duct and surrounding organs is a rare complication of IPNB with only few case reports in the literature (8–14) (**Table 1**). Clinical manifestations of fistula include right upper quadrant pain, jaundice, recurrent cholangitis and sepsis. The pathogenesis of fistula formation into other adjacent organs may be high pressure caused by mucin-filled bile ducts and local inflammation or direct tumor invasion of IPNB (11, 21). The histopathological exam disclosed cholangiocarcinoma in the fistula tract in our patient, demonstrating direct tumor-related transmural invasion of stomach wall. Compared with pancreatic intraductal papillary mucinous neoplasm, IPNB with fistula formation is relatively rare, probably because autodigestion by enzyme-rich pancreatic juice also plays a crucial role in fistula formation of pancreatic intraductal papillary mucinous neoplasm (21).

**Table 1:** Reported Cases of IPNB with Fistula Formation.

Reference	Year	Sex	Age	Location	Pathology	Fistula connection	Proposed Mechanism
Ohtsubo et al. (8)	1999	F	78	IHD	Biliary papillomatosis	Stomach	NA
Kim et al. (9)	2011	F	63	CBD	IPNB	Duodenum	Mechanical penetration
Barresi et al. (10)	2012	M	53	Left IHD, CHD	IPNB with cholangiocarcinoma	Duodenum	Tumor invasion
Hong et al. (11)	2014	F	87	Left IHD, CBD	IPNB with low grade dysplasia	Stomach, duodenum	Mechanical penetration
Liu et al. (12)	2018	F	66	IHD, CBD	NA	Duodenum	Mechanical penetration
Ren et al. (13)	2019	M	52	CBD	IPNB with microinvasive cholangiocarcinoma	Pancreas	Mechanical penetration
Terasaki et al. (14)	2020	F	79	B3, CBD	IPNB with cholangiocarcinoma	Stomach	Mechanical penetration
Current study	2023	M	72	Left IHD	IPNB with cholangiocarcinoma	Stomach	Tumor invasion

IHD, intrahepatic duct; CHD, common hepatic duct; CBD, common bile duct; B3, liver segment 3 IHD; tumor invasion, invasive tumor found at fistula tract on histopathology; mechanical penetration: mechanical penetration by mucin-filled ducts; NA, not available.

There are variable radiological presentations of IPNB, with respect to different tumor location, degree of mucin production, size and morphological features of intraductal tumors (22). In general, the imaging findings can be classified into four types: masses with proximal ductal dilatation, disproportionate ductal dilatation without masses, cystic lesion, and masses with proximal and distal ductal dilatation (19, 22). The imaging finding of our patient is consistent with intraductal masses with proximal and distal ductal dilatation complicated with hepatogastric fistula. Cross-sectional imaging such as CT and MRI can demonstrate accurate delineation of bile duct dilatation, presence of intraductal mural nodules or cholelithiasis, as well as invasion of adjacent organs. Differentiating papillary tumors and mucobila may be challenging. The combination of contrast enhanced studies and diffusion-weighted imaging may aid in differential diagnosis (23). Endoscopic retrograde cholangiopancreatography (ERCP) is a relatively invasive exam compared to CT and MRI but has the advantage of obtaining histological or cytological diagnosis.

Surgery is the potential curative treatment for IPNB. To ensure proper staging, most patient will require an (extended) hemi-hepatectomy and lymphadenectomy involving at least six locoregional lymph nodes (4). A recent systematic review and meta-analysis demonstrated that up to 43% of resected specimen of IPNB contains invasive cholangiocarcinoma, and the prognosis is worse in patients with invasive component (5). Even so, the intraductal papillary type of cholangiocarcinoma has a significantly more favorable outcome compared to periductal-infiltrating and mass-forming type cholangiocarcinoma (24, 25). Therefore, achieving a complete margin-negative (R0) resection, along with a sufficient future liver remnant is the primary objective of surgery and significantly improves patient outcome (24, 26, 27). Additionally, a recent European multicenter observational study (EUR-IPNB study) revealed achieving a textbook outcome (non-prolonged hospital stay, absence of any ≥ Clavien-Dindo grade III complication, readmission, or mortality within 90 postoperative days) was an independent prognostic factor of favorable overall survival (27). Nowadays, with the advancement of endoscopic treatment, stent insertion, mucin suction or lavage may play a role in patients who are not surgical candidate due to medical conditions (11).

## Conclusion

In conclusion, we report a rare case of IPNB with invasive cholangiocarcinoma, leading to direct transmural tumor invasion of stomach with hepatogastric fistula formation. Accurate preoperative imaging evaluation and thorough intraoperative cholangioscopy evaluation are key to curative resection.

## Data availability statement

The original contributions presented in the study are included in the article/supplementary material. Further inquiries can be directed to the corresponding author.

## Ethics statement

The studies involving human participants were reviewed and approved by ethics committee of Chang Gung Memorial Hospital at Linkou. The patients/participants provided their written informed consent to participate in this study.

## Author contributions

W-HC researched the data and wrote the manuscript. R-CW and T-CC evaluated the pathological results and provided figures for this article. T-CC, H-KL and C-HL contributed to the discussion. S-YW and C-NY guided the writing ideas and reviewed the manuscript. All authors agree to be accountable for the content of the work. All authors contributed to the article and approved the submitted version.
